# National screening for Egyptian children aged 1 year up to 12 years at high risk of Autism and its determinants: a step for determining what ASD surveillance needs

**DOI:** 10.1186/s12888-023-04977-5

**Published:** 2023-06-28

**Authors:** Ammal M. Metwally, Mona A. Helmy, Ebtissam M. Salah El-Din, Rehan M. Saleh, Ehab R. Abdel Raouf, Ali M. Abdallah, Zeinab Khadr, Amal Elsaied, Mostafa M. El-Saied, Randa I. Bassiouni, Dina A. Nagi, Manal A. Shehata, Inas R. El-Alameey, Hazem M. El-Hariri, Somia I. Salama, Thanaa M. Rabah, Ghada A. Abdel-Latif, Lobna A. El Etreby, Dalia M. Elmosalami, Samia M. Sami, Eman Eltahlawy, Nihad A. Ibrahim, Nahed A. Elghareeb, Hala Y. Badawy, Eman M. Dewdar, Engy A. Ashaat

**Affiliations:** 1 Community Medicine Research Department/ Medical Research and Clinical Studies Institute, National Research Centre, Cairo, 60014618 Dokki Egypt; 2 Environmental and Occupational Medicine Department, Environmental and Climate Change Research Institute, National Research Centre, Dokki, Cairo, 60014618 Egypt; 3 Child Health Department/ Medical Research and Clinical Studies Institute, National Research Centre, Dokki, Cairo, 60014618 Egypt; 4 Child With Special Needs Dept./ Medical Research and Clinical Studies Institute, National Research Centre, Dokki, Cairo, 60014618 Egypt; 5 Quantitative Methods Department - Aswan University, Tingar, Egypt; 6 Department of Statistics, Faculty of Economics and Political Science, Cairo University, Giza, Egypt; 7 The Social Research Center of the American University in Cairo, Cairo, Egypt; 8 Clinical Genetics Dept./ Human Genetics and Genome Research Institute, National Research Centre , Dokki, Cairo, 60014618 Egypt; 9 Disability Prevention General Directorate, Ministry of Health and Population, Cairo, Egypt

**Keywords:** Autism spectrum disorder, Egyptian national prevalence, Socio-demographic, Epidemiological characteristics, Perinatal

## Abstract

This study aimed to provide a national estimate of the prevalence of the high risk of autism spectrum disorder (ASD) and their determinants. A national screening survey was conducted for 41,640 Egyptian children aged 1 to 12 years in two phases. Tools used were Vineland's Adaptive Behavior Scales, Modified Checklist for Autism in Toddlers, Gilliam Autism Rating scale, and Denver II Developmental screening test. The overall prevalence of children at high risk of ASD was 3.3% (95% CI:3.1%–3.5%). Children living without mothers in homes, suffered from convulsions (AOR = 3.67; 95%CI:2.8–4.8), a history of cyanosis after birth (AOR = 1.87; 95% CI:1.35–2.59) or history of LBW babies (AOR = 1.53; 95% CI:1.23–1.89) carried higher odds of being at high risk of ASD.

**Supplementary Information:**

The online version contains supplementary material available at 10.1186/s12888-023-04977-5.

## Introduction

According tothe Diagnostic and Statistical Manual of Mental Disorders (5th edition) (DSM-5) criteria, autism spectrum disorder (ASD) is defined as a group of neurodevelopmental disorders characterized by early-onset and persistent impaired social interaction, communication, and restricted/repetitive behavior and interest [22, 43, 96]. Having a child with ASD is accompanied by a great burden of care on family members; physically, emotionally, and financially [7, 57].

The Global Burden of Disease Study estimated ASD prevalence and years living with disability among the common six prevalent developmental disabilities in children under the age of five years [73]. The prevalence of ASD has increased considerably in the last two decades, reaching around 1–2% of the general population due to greater recognition and knowledge of the disease [12].

Around 52 million children with ASD were recorded worldwide in 2010, with a prevalence of 7.6 per 1000 [88]. In 2018, the Centers for Disease Control and Prevention (CDC) reported that nearly 1 in 59 children had ASD. In 2020, that number increased to 1 in 44 children [19]. The most recent data from Europe and the United States show a significant increase in the measured prevalence of autism over the last two decades from 0.48 to 3.13 percent [66]. However, research on ASD prevalence in Arab countries was limited to high-income countries; Systematic meta-analysis in these countries revealed wide variations, Oman, the United Arab of Emirates, Saudi Arabia, Bahrain, Kuwait, and Qatar [1, 8]. Meanwhile, there is a shortage of data concerning ASD prevalence in developing Arab countries. Generally speaking, the great variation in the prevalence of ASD estimates that was found in Arab countries was due to the variation in the study methods, age of participants, case ascertainment approaches, and diagnostic tools used as there is no standardized method for screening of ASD until now which is considered a challenging situation. Due to the same reasons, studies in Egypt estimated great variability of ASD prevalence ranging from 5.4/1000 [102] to 33.6% [85]. At the same time, studies in Egypt were limited to, confined areas, facility-based, and with a limited sample size [41, 102].

Till now, the causes and risk factors of ASD are not well known with many operational risk factors; prenatal, perinatal, and environmental [87, 97]. Even though, there is growing evidence that ASD has become a public health problem in many countries. Such findings indicated that autism has to be perceived as an important problem that deserves to be in the light spot for policymakers to allow delivery of targeted services, especially in developing countries where access to therapeutic effective services is either limited or not existed. Early detection of ASD is crucial for prompt and early intervention to achieve significant improvement in behavioral performance and the intellectual abilities of children with ASD.

This study aimed at providing a national screening estimate of children who are at high risk of ASD among Egyptian children aged 1 to 12 years with integration of their developmental level based on reliable screening tools used during two phases. The study identified the possible determinants of the at high risk of ASD that are related to sociodemographic, epidemiological, maternal, and child health status.

## Methodology

### Study type, design, and setting

The current study was both community and facility based, represented a part of a national Egyptian survey for estimation of the prevalence of autism spectrum disorder among children aged 1 to 12 years. This survey included four phases. Phase one was a household community based survey planned for detection of children with deviated development and potentially suspicious for autism. In phase two, these children were referred to maternal and child health centers and screened for ASD using ASD screening tools. Diagnosis of ASD was confirmed in phase three using DSM-5 criteria and CARS. Children with confirmed diagnosis of ASD were assessed for intellectual functions and adaptive behavior in phase four.

The current study reported results of the first and second phases to detect the at high risk of autism, meanwhile confirmed cases with autism and their characteristics will be published in another manuscript. It was a cross-sectional national screening study which was done in two phases. The study was conducted in 8 Governorates representing all geographic regions of Egypt according to their population density. The whole study was conducted over 24 months starting from December 2017 till December 2019.

### Target group

The study targeted parents or caregivers with their children aged 12 months up to 12 years at the visited houses.

#### Inclusion and exclusion criteria

Any child in the age range of 12 months to 12 years whether previously identified as having ASD or with typical or deviated development according to  [15, 99], whose parents accepted to participate was included in the study. The response rate of acceptance for participation was 100%.

Exclusion criteria: Children with known or previously diagnosed with genetic disorders (e.g., Down syndrome, Turner syndrome, or fragile-X syndrome) as assessed by a clinical genetics team from the National Research Centre of Egypt. The validated WHO ten-question screening tool with their probes was explained to the household for detecting any disability that is affecting hearing, vision, movement, learning, thinking, or social relationships whether for children or their parents [24, 70].

The researchers ensured that all children with detected disabilities or delay other than autism were enrolled in rehabilitation programs by the Ministry of Health. Those with autism were referred for management to the outpatients' clinics of Children with Special Needs and clinic of Clinical Genetics Department of the National Research Centre of Egypt.

### Sampling frame and cluster preparation

Three sampling frames were chosen in three stages; the first sampling frame used was the comprehensive list of the governorates in Egypt according to the enumeration census from the Central Agency for Public Mobilization and Statistics (Central Agency for Public Mobilization and Statistics (CAPMS) [20]. In the first stage, a representative sample of 8 governorates was selected to represent the main geographic areas in Egypt including; One urban (Cairo), three Upper Egypt (Fayoum, Assuit, and, Aswan), three Lower Egypt (Damietta, Dakahlia, and, Gharbia) and One border -Frontier- (Marsa Matrouh). In the second stage, a representative sample of cities and local units were selected from each governorate. In this selection process, each governorate was divided into three categories according to its human development scores, namely low, medium, and high [10, 26]. One city for urban areas and a local unit for rural areas were selected from each category for each governorate. In the third stage, for urban areas, the selected cities were divided into city blocks then choosing one or two blocks for surveying. For the rural areas, one or two villages were selected from the selected local units (according to population size). A total of 45-blocks ensured both the adequate sample size and heterogenicity of data collected. In this stage, households in the selected cities and villages blocks were screened. The sample was allocated to be proportional to the size of large governorates. While governorates with relatively small populations were assigned to arbitrary sample sizes with adjusting weights during the analysis of the data.

### Survey sample size

The sample size calculation is based on the estimated prevalence of ASD which was 1% as indicated from the previously stated survey studies in Arab countries [85]. Taking this prevalence into consideration ensured the largest sample size at a level of accuracy set at 0.0049 (margin of error), confidence limit of 95%, and the least reliable of the questionnaires used for detecting Autism was 80%. Accordingly, targeting 40,000 children were expected to ensure data accuracy for the provision of estimates of the prevalence of ASD [44]. The actual number obtained was 41,640 from 22,026 surveyed houses along 45 blocks from the eight targeted governorates.

### Study instruments

Autism spectrum disorder is characterized by sorts of delay and deviation in the development of social, communication, and cognitive functions, which begin in the first years of life. Autism spectrum disorder has a varied range of syndrome expression and its diagnosis presents actual challenges [94]. In the current study assessment of the targeted children was done on two levels.

#### 5.1First phase screening

This phase was a screening phase carried out at the household level. It was directed to parents/caregivers of children in the age range of 12 months to 12 years. Due to lack of professional specialists for ASD screening of the enormous number of children (41,460 children), and lack of registration of developmentally delayed children in Egypt, the research team preferred to use the Arabic version of Vineland Adaptive Behavior Scales, (VABSA) [6] for assessment in the first phase screening. The main output of this phase was children with deviated development or aberrant behavior who potentially had one of the neurodevelopmental disorders among which ASD was suspected. Through face-to-face interviews with the parents or caregivers, three parts of a household-developed questionnaire were fulfilled.

#### The first part of the household questionnaire

Collected information on housing and sociodemographic characteristics including age, sex, birth order, number of siblings, maternal age, residence, and parental education and occupation  [10, 26, 31].

#### The second part of the questionnaire

collected information about the epidemiological, maternal, and neonatal risk factors for autism that were identified before [37].

#### The third part of the questionnaire

Included adaptive behavior assessment using VABS.

The Vineland Adaptive Behavior Scales (VABS) is one of the most commonly used measures of parent-reported adaptive behavior for individuals from birth through adulthood [92]. It measures adaptive functioning across four domains: Communication, Daily Living Skills, Socialization and Motor Skills. It supports diagnosis of intellectual and developmental disabilities, autism, and developmental delay [42]. The measured four domains integrated eleven subdomains. For example, the subdomains of socialization domain inscribe interpersonal relationships, play and leisure time, and coping skills which are greatly impacted in children with ASD. A number of old and recent studies suggested the usefulness of VABS as a well-standardized, normative assessment instrument for verifying autistic social dysfunction [95, 34, 90, 30, 83].

The Vineland survey form, which is administered in 15–20 min by a trained interviewer, was used in this study. The mean of the adaptive behavior composite score is 100 with SD of 15. A delay in specific domain is considered if the score of that domain is 2 SD below the mean (< 70). The VABS survey form demonstrated high reliability coefficient ranging from 0.83 to 0.94 across all domains [92]. Discovered children with low scores (< 70) on VABS suggesting developmental delay were referred to maternal and child health centers and screened for ASD using ASD screening tools.

#### 5.2Second phase screening

In this phase the referred children were screened for ASD according to their ages. Either with the Modified checklist for autism in toddlers-revised [68, 76] for children aged 1–3 years. Or with (Gilliam Autism Rating scale (GARS-2) for children aged 3–12 years [82]. Denver II Developmental screening test (DDST) was used for developmental assessment and developmental regression of children up to 6 years [33]. The output of this phase was to find out the children who were scored positive for autism according to M-Chat, and GARS-2.

- The M-CHAT is a parent-completed questionnaire that includes items from the CHAT that cover a broader range of signs and a wider age range (16–30 months). The sensitivity of the M-CHAT was reported to be as high as 85%, but specificity is low around 40%. Parents were asked in more detail about symptoms identified by the first phase questionnaire. This interview increases the specificity of the M-CHAT and is highly recommended. If the total score of M-CHAT was 0–2, The child has screened negative. Rescreening is needed at 24 months if the child is younger than 2 years old. If the total Score was 3–7: The child is at moderate risk, and at a score of 8–20 the child is at high risk. In cases of moderate and high-risk individuals, the child should be referred for diagnostic evaluation and early intervention. In the current study, the child was referred for diagnostic evaluation by the NRC consultants when the total score was 3 or more [56].
- Gilliam Autism Rating Scale (GARS-2) although could be used for verification of autism diagnosis it was used as screening for children aged 3–12 years who have severe behavioral problems that may be indicative of autism. GARS-2 is a 42-item norm-referenced screening tool to help professionals identify ASD. The GARS-2 gathers information about specific characteristics typically noted in children with autism spectrum disorders in three subscales (Stereotyped Behaviors, Communication, and Social Interaction) and it contains a developmental history. The Autism Index is calculated by first calculating the raw scores of each subscale and then converting them into derived standard scores. Scores of 85 or higher on the Autism Index indicate that an individual is likely to have autism. Scores of 70 to 84 indicate that an individual may have autism, and any score of 69 or less suggests that it is unlikely that the individual has autism [35]. In the current study, nurses were instructed to refer children to consultants once the autism index was 70 or more to avoid missing probable cases.
- Denver II Developmental Screening Test (DDST): This test is a screening measure to identify possible developmental delays in children ages birth through six years. Screening includes four developmental areas: personal-social, fine-motor adaptive, language, and gross motor. There is one form for all ages. The test includes direct child assessment and parent report, and takes 10–20 min to complete. Each item is scored as passed, failed, or refused. DDST is a norm-referenced test. Items that can be completed by 75% of children but are failed by the examined child are called cautions; items that can be completed by 90% of children but are failed by the examined child are referred to as delays. The test is interpreted as Normal, Suspect, or Untestable according to the number of items upon which the child scores below or within the expected age range [33]. The sensitivity and specificity of this test are 0.83 and 0.43 respectively [36]. In the current study, all children interpreted as Suspect or Untestable were referred to specialized physicians in the health care centers of the Ministry of Health and Population (MOHP) and NRC team to ascertain the results of screening test.

### Survey implementation

Before the implementation of phase one and two, condensed training sessions about how to conduct the relevant used questionnaires in a standardized way were done. Both phases were done under the supervision of specialized team members.

Phase one of the survey was conducted by the pre-trained 64 social workers (average 6/governorate) under the supervision of a collaborative team from the Cairo Demographic Center (CDC) with professional team members from the National Research Centre of Egypt (NRC).

The range of the targeted houses per governorate was from 1960–4170 (S table-1). Each social worker targeted an average of 6 houses per day for an average of 5 months. The implementation of both phase one and phase two was conducted over one year in a simultaneous way along the geographical areas of the eight randomly selected governorates. A pilot study was performed on 80 participants (10/governorate) to ensure the validity of the questionnaire items through revising and modifying difficulty- understood items or language and then re-introduced them.

In this phase, Vineland Adaptive Behavior survey form was administered by the social workers who have properly trained on this questionnaire. It was reported that, a psychologist, social worker, or other professional who has appropriate training in interview techniques can complete these forms [72].

Before starting implementation of phase II, 208 selected nurses were trained by specialized consultants (26 per each governorate) to ensure using the screening tests for autism and development and calculate the score. This number was chosen for covering 45-blocks of Shiaka and villages (within 24 Kism in urban areas and 21 markaz in rural areas) to ensure heterogeneity of the data collected (S table-1). For suspicious children who did not attend the health facilities, outreach visits by the trained nurses were scheduled for meeting the children and undergoing reliable tests. Moreover, recognized children with suspected developmental delays and/or autism were confirmed by specialized physicians in the health care centers of the Ministry of Health and Population (MOHP) and NRC to ascertain the results of the 2nd screening phase.

In this phase the well-trained nurses administered 3 psychological tests:

The M-CHAT and Denver Developmental screening tests (DDST). Administration of these tests usually does not need a specialist and can be administered by individuals who have trained to proficiency [16, 38, 56]. Nurses were directed to refer children to consultants when the DDST was interpreted as suspect or untestable and when the child had a score of 3 or more on M-CHAT.

Gilliam Autism Rating Scale (GARS-2), although administration of this test needs a professional individual, it was administered by the well-trained nurses who have received extensive training and were closely supervised by qualified consultants and supervisors. Nurses were instructed to refer children to consultants once the autism index was 70 or more to avoid missing probable cases. (The child is likely to be diagnosed with autism at scores of 85 or more).

### Statistical analysis

Data were analyzed using Statistical Package for the Social Sciences (SPSS) version 22.0 software (IBM SPSS Statistics for Windows, Version 22.0. Armonk, NY: IBM Corp.). All data were represented by percentages and comparisons between groups were done using crude odds ratios and 95% confidence intervals (CI) were calculated in comparison between DDs and children without delays. Probability values (*p*) < 0.05 were regarded as statistically significant. Logistic regression analysis was done to assess the contribution of each independent variable to predict the odds of ASD based on the values of the independent variables (risk factors for ASD) by using the Adjusted odds ratio [93]. A significant association is considered if the 95% CI does not include the value of 1.0, and a cutoff*p*-value of less than 0.05 is used for all tests of statistical significance in this study.

## Results

Table 1 shows the socio-demographic characteristics and perinatal problems of the study population. The parents or the caregivers of 41,640 children in the age range 1–12 years were participated in the study. Children were slightly insignificantly higher in rural than urban communities. They were equally distributed among social classes. They were distributed among the randomly selected governorates according to their population size. The surveyed boys were insignificantly slightly higher than girls (51.5% versus 48.5% respectively). Half of the children were in the age group 6 to 12 years (48.8%). The least percentage of children were in the age group 1- < 3 years (20.1%). 95.8% of children above 6 years went to school. The mean age at which mothers gave birth was 26.06 ± 6.13 years and their current mean age was 32.04 ± 6.81 years. Regarding education levels, almost half of mothers and fathers completed their higher school degrees (44.7% and 44.2% respectively). Most of the mothers were unemployed (84.8%). Houses without mothers were 0.7% versus 4.6% without fathers. Among the perinatal conditions, the presence of a history of neonatal jaundice after birth was the most prevalent (26.5%) followed by a history of difficult labor (14.6%). 0.9% of houses had mothers who were physically or mentally disabled and 4.3% had twins. History of LBW was reported among 4.4% versus 1% with a history of premature delivery.

**Table 1:** Characteristics of the study population

**Socio-demographic characteristics**	**Surveyed children *n* = **(41,640)
	N (%)
**Locality (Urban/ Rural)**	
Urban	19,422 (46.6%)
Rural	22,218 (53.4%)
**Social class**	
Low	13,586 (32.6%)
Middle	13,887 (33.4%)
High	14,167 (34.0%)
**Geographical Distribution**	
Cities	6919 (16.6%)
Lower Egypt	15,892 (38.2%)
Upper Egypt	14,344 (34.5%)
Frontier	4485 (10.8%)
Mean no of households ± SD	4.97 ± 1.281
**Sex**	
Surveyed boys	21,437 (51.5%)
Surveyed girls	20,203 (48.5%)
**Age**	
1- < 3 years	8383 (20.1%)
3- < 6 years	12,933 (31.1%)
6–12 years	20,324 (48.8%)
**School attendance status**^a^** (*n* = 20,332)**	
Child goes to school	19,470 (95.8%)
Does not go to school	858 (4.2%)
Mean Mother age at giving birth ± SD	26.06 ± 6.13
Mean Current maternal age ± SD	32.04 ± 6.81
**Mothers Education**	
Illiterate/ below high school	16,046 (38.5%)
High School	18,609 (44.7%)
University or higher	6674 (16.0%)
**Fathers Education**	
Illiterate/ below high school	14,666 (35.2%)
High School	18,390 (44.2%)
University or higher	6662 (16.0%)
**Mothers´ work**	
Employed	6014 (14.4%)
Unemployed	35,315 (84.8%)
**Presence of mothers or fathers**	
No father in the HH	1922 (4.6%)
No mother in the HH	299 (0.7%)
**Prenatal, natal and neonatal problems**	
History of Premature delivery (< 37 weeks gestation)	413 (1%)
History of Low birth weight (LBW) (< 2500 g)	1848 (4.4%)
Children with history of jaundice after birth	11,028 (26.5%)
Children with history of cyanosis after birth	546 (1.3%)
**Children with history of any convulsions after birth**	**675 (1.6%)**
Children kept in an incubator for more than two days	3078 (7.4%)
Children with history of meningitis after birth	378 (0.9%)
Twins	1774 (4.3%)
Mothers have any health problem during pregnancy^b^	2770 (6.6%)
Difficult labor^c^	6092 (14.6%)
Disabled mothers^d^	373 (0.9%)

^a^of children aged 6 -12 years,

^b^Mothers having complications during pregnancy such as gestational diabetes, hypertension, iron deficiency anemia, anxiety, depression, or infection [18]

^c^Difficult labor refers to prolongation in the duration of labor, especially in the first stage of labor. It can be a contributor to maternal mortality and morbidity if unrecognized or untreated [13]

^d^disabled mother: physically or mentally disabled; Hearing, Vision, Mental, Movement, Speech [99, 100]

**Fig. 1:**
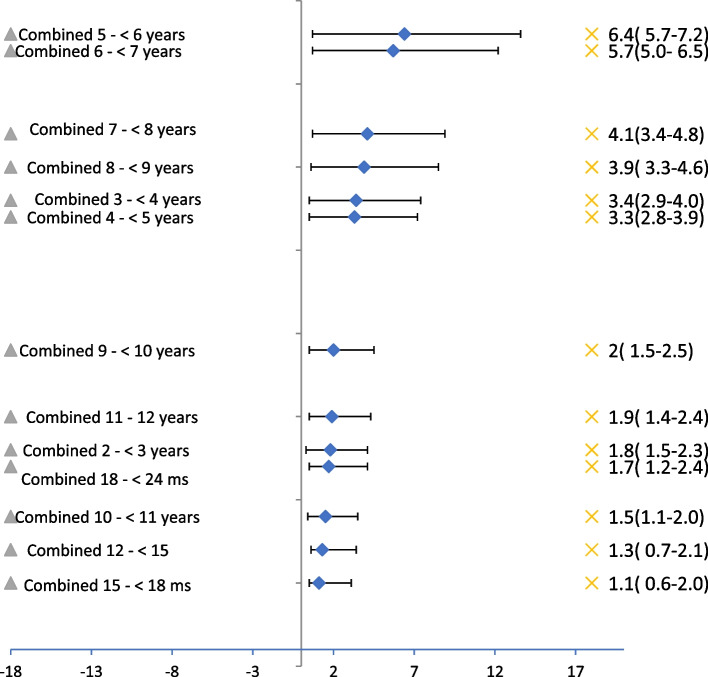
Screening plot per age distributed according to the highest prevalence/age, the overall prevalence among children aged 1–12 years (Combined sex) = 3.3% (95% CL: 3.1%-3.5%), Population Survey

**Fig. 2:**
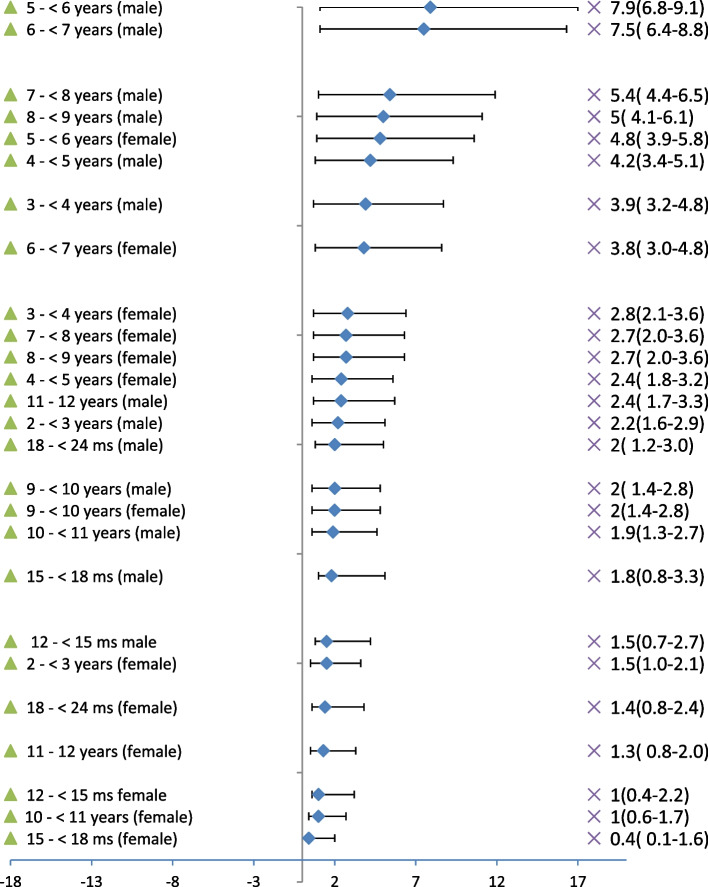
Screening plot per sex distributed according to the highest prevalence/sex, COR males/Females = 1.72 (95% CL: 1.53–1.92), Population Survey

Age and sex-specific estimates of ASD appeared in Figs. 1 and 2 respectively. Group-specific prevalence was highest for the age group 5- < 6 years (6.4%, 95% CI: 5.7%–7.2%) followed by age group 6- < 7 (5.7%, 95% CI: 5.0%–6.5%) as shown in Fig. 1.

The prevalence of ASD among boys was almost double that of the girls for these two specific age groups (COR = 1.70, 95% CI: 1.32%–2.19% & COR = 2.06, 95% CI: 1.54%–4.88% respectively) as shown in Fig. 2.

Summing the prevalence of the at high risk of ASD among children aged 1–12 years was 3.3% (95% CI: 3.1%–3.5%). The prevalence of the at high risk of ASD among boys aged 1–12 years was 4.1% (95% CI: 3.4%–4.8%) versus 2.4% (95% CI: 1.7%–3.3%) among girls in the same age group. Having a child at high risk of ASD was likely to be found among boys one and three-quarters times that of girls in the age range 1–12 years (COR 1.72, 95% CI: 1.53%–1.92%).

Table 2 shows the odds of having features of autism among surveyed children according to the socio-demographic and child characteristics. Having at high risk of ASD was higher among children belonging to the middle class than belonging to either the high and low classes (COR 1.53, 95% CI: 1.34–1.73 & COR 1.72, 95% CI: 1.51–1.97 respectively). Living in urban communities was associated with significantly increased odds of at high risk for ASD than living in rural (COR = 1.71, 95% CI: 1.53–1.91). Living in Lower Egypt or in Frontiers significantly decreased the odds of at high risk of ASD than living in cities by more than 60% (COR = 0.39, CI: 0.34–0.44 & COR = 0.36, CI: 0.29–0.45 respectively).

**Table 2:** Odds of having at high risk of Autism among surveyed children according to the sociodemographic and child characteristics

**sociodemographic and child characteristics**^a^	**All ASD** **(*n* = 1370)** **n(%)**	**Boys** **(*n* = 879)** **n(%)**	**Girls** ***n* = 491** **n(%)**	**COR (95% CI)** **All versus Healthy**
**Locality (Urban/ Rural)**				
**Urban (**19,422)	815 (4.2)	513(2.65)	302(1.6)	Urban/ rural 1.71* (1.53–1.91)
**Rural (**22,218)	555 (2.5)	366(1.65)	189(0.85)	
**Social class**				
**Low (**13,586)	351 (2.6)	243 (1.8)	108(0.8)	Middle vs. low 1.72(1.51–1.97)**
**Middle (**13,887)	607 (4.4)	361 (2.6)	246 (1.8)	Middle vs. high 1.53(1.34–1.73)**
**High (**14,167)	412 (2.9)	275 (1.9)	137(1.0)	low vs. high 0.89(0.766–1.02)
**Geographical Distribution**				
**Cities (**6919)	462 (6.7)	285(4.1)	177(2.6)	Lower vs Cities 0.39 (0.34–0.44)**
**Lower Egypt** (15,892)	428 (2.7)	264(1.7)	164(1.0)	lower vs. Frontiers 1.07 (0.87–1.32)
**Upper Egypt (14,344)**	367 (2.6)	251(1.7)	116(0.9)	upper vs. Frontiers 1.02(.82–1.26)
**Frontier (4485)**	113 (2.5)	79(1.8)	34(0.7)	Frontiers vs cities 0.36(0.29–0.45)**
**Age category**				
**1- < 3 years (**8383)	137 (1.6)	86 (1.0)	51 (0.6)	3- < 6 years vs. 1- < 3 2.77(2.29–3.34)**
**3- < 6 years (**12,933)	568 (4.4)	360(2.8)	208(1.6)	3- < 6 years vs. 6–12 years 1.46(1.21–1.52)**
**6–12 years (**20,324	665 (3.3)	433(2.1)	232(1.2)	6-12 years vs. 1- < 3 years 0.99 (0.89–1.1)
**School attending status**^b^** (*n* = 20,332)**				
**1. Child goes to school (19,470)**	1247 (6.4)	790 (4.1)	457 (2.3)	2 vs 1 5.2 (4.43–6.17)**
**2. Does not go to school (858)**	123 (14.3)	89(10.4)	34(3.9)	

^a^percent was calculated out of rows

^b^of children aged 6 -12 years

^*^p-value significant at < 0.05

^**^p-value highly sig at < 0.01

Children aged 3 – < 6 years were the most likely to be at high risk of ASD with the highest prevalence (4.4%) with significantly twice and three quarters more odds than those of the age group 1- < 3 years (COR = 2.7, CI: 2.29–3.34) and one and a half more odds than children aged 6–12 years (COR = 1.46, CI: 1.21–1.52).

Non-school attendance was significantly five times more among children with at high risk of ASD (COR = 5.2, 95% CI: 4.43–6.17) than non-autistic children.

Concerning the odds of having at high risk of ASD among surveyed children according to the maternal and paternal characteristics (Table 3), the age of mothers who gave birth above 35 years was associated with significantly increased odds of ASD than mothers giving birth below 18 years by one and a half-time (COR = 1.57, 95% CI: 1.13–2.18).

**Table 3:** Odds of having at high risk of Autism among surveyed children according to the maternal and paternal characteristics

**maternal and paternal characteristics**^a^ ***N* = 41,640**	**All ASD** **(*n* = 1370)** **n(%)**	**Boys** **(*n* = 879)** **n(%)**	**Girls** ***n* = 491** **n(%)**	**COR (95% CI)** **All versus Healthy**
**Mother age at giving birth**				
** < 18 (**1976)	**50 (2.5)**	38(1.9)	12(0.6)	** > 35 vs. < 18** 1.57(1.13–2.18)*
**18 to < 35 (**35,958)	**1167 (3.2)**	752(2.1)	415(1.1)	**18- < 35 vs. < 18** **1.29(0.97–1.72)**
** > 35 (**3407)	**133 (3.9)**	80(2.9)	53(1.7)	**18- < 35 vs. > 35** **.83(0.69–1.0)**
**No mother (**299)	**20 (6.7)**	9(3.0)	11(3.7)	**No mother vs. mother at home** 2.04 (1.29–3.23)**
**Current Mothers Age**				
** < 35 (*n* = 36,344)**	**1179 (3.2)**	761(2.0)	418(1.2)	** = > 35 vs. < 35** **1.08(0.97–1.21)**
** = > 35 (*n* = 5296)**	**191 (3.6)**	118(2.2)	73(1.4)	
**Mothers Education**				
**1. Illiterate/ Read & write to Prep (16,046)**	580 (3.6)	369(2.2)	211(1.4)	**3 vs. 1** 0.66(0.55–0.79)**
**2. High School & technical (18,609)**	628 (3.4)	416(2.2)	212(1.2)	**2 vs. 1** **0.90(0.80–1.01)**
**3. University or higher (6674)**	**142 (2.1)**	85(1.2)	57(0.9)	**2 vs. 3** 1.59(1.32–1.9)*
**Fathers Education**				
**1. Illiterate/ Read & write to Prep (14,666)**	**569 (3.9)**	377(2.6)	192(1.3)	**3 vs. 1** 0.61(0.51–0.72)*
**2. High School & technical (18,390)**	**569 (3.0)**	367(2.0)	202(1.0)	**2 vs. 1** 0.75(0.67–0.85)*
**3. University or higher (6662)**	**133 (2.0)**	77(1.2)	56(0.8)	**2 vs. 3** 1.55(1.28–1.8)*
**4. No father at home (1922)**	**99 (5.2)**	58(3.0)	41(2.2)	**No Father vs. Father at home** 1.61(1.3–1.98)*
**Mothers´ work**				
**1. Work (paid-unpaid-her own) (*n* = 6014)**	**196 (3.3)**	121 (2.0)	75 (1.3)	**COR 2 vs 1** **1.00(0.86–1.17)**
**2. Unemployed (35,315)**	**1154 (3.3)**	749(2.1)	405 (1.2)	

^a^percent was calculated out of rows

^*^p-value significant at < 0.05

^**^p-value highly sig at < 0.01

Children with mothers or fathers who had higher education were less likely to have at high risk of ASD, especially those who had a college or greater education level with a fewer odds of a range from 25 to 39% than lower grades of education. The influence of higher paternal education was higher than that of maternal education. Mothers’ work or current age did not show any association with having at high risk of ASD.

Whereas living without mothers was associated with two times increased odds for at high risk of ASD (COR = 2.04, 95% CI: 1.29–3.23) than living with mothers. Living without fathers carries significantly one and a half odds for at high risk of ASD than the presence of fathers (COR = 1.61, 95% CI: 1.30–1.98).

The odds of having at high risk of ASD among surveyed children according to all the studied medical perinatal history problems were shown in Table 4. All the studied medical histories for both mothers and children were risk factors for at high risk of ASD. The odds for at high risk of ASD are largely affected by the type of medical perinatal history.

**Table 4:** Odds of having at high risk of Autism among surveyed children according to the medical perinatal history problems and postnatal child problems

**Perinatal and postnatal problems**^a^ ***N* = 41,640**	**All ASD** **(*n* = 1370)** **n(%)**	**Boys** **(*n* = 879)** **n(%)**	**Girls** ***n* = 491** **n(%)**	**COR (95% CI)** **All versus Healthy**
Maternal problems				
Disabled mothers (apparent disability) (*n* = 373)	26 (7.0)	12 (3.1)	14 (3.9)	7.77 (5.9–10.1)**
Mothers having any health problem during pregnancy (*n* = 2770)	215 (7.8)	140 (5.1)	75 (2.7)	2.75 (2.36–3.20) **
Difficult labor (*n* = 6092)	309 (5.1)	195 (3.2)	114 (1.9)	1.74 (1.53–1.98) *
Perinatal problems				
Child suffer from any convulsions (*n* = 675)	132 (19.6)	89(13.2)	43(6.4)	7.80 (6.40–9.51) **
Child suffer from cyanosis after birth (*n* = 546)	91(16.7)	55(10.1)	36(6.6)	6.23 (4.94–7.85) **
Child suffer from meningitis (*n* = 378)	49 (13)	29(7.7)	20(5.3)	4.50 (3.32–6.12) **
Twins (*n* = 1774)	57 (3.2)	39 (2.2)	18(1.0)	3.75 (3.18–4.41) **
Child born less than 7 months (preterm delivery) (*n* = 413)	41 (9.9)	29 (7.0)	12 (2.9)	3.31 (2.39–4.59) **
LBW (less than 2.5 kg at birth) (*n* = 1848)	156 (8.4)	94 (5.1)	62(3.3)	2.93 (2.46–3.49) *
Child kept in an incubator for more than two days (*n* = 3078)	241 (7.8)	150(4.9)	91(2.9)	2.82 (2.44–3.25) *
Child suffer from jaundice after birth (*n* = 11,028)	524 (4.8)	343(3.1)	181(1.7)	1.76 (1.57–1.96)*

^a^percent was calculated out of rows

^*^p-value significant at < 0.05

^**^p-value highly sig at < 0.01

Mothers with apparent physical or mental disabilities carried the highest odds for the at high risk of ASD among the other maternal problems (COR = 7.77, 95% CI: 5.9–10.1). Whereas, children with a history of cyanosis after birth (COR = 6.23, 95% CI: 4.94–7.85) or convulsions (COR = 7.8, 95% CI: 6.40–9.51) or had a history of meningitis after birth (COR = 4.50, 95% CI: 3.32–6.12) were the most of postnatal child problems for carrying the highest odds for the at high risk of ASD than being a healthy child.

The odds of presenting features of Autism among surveyed children according to sex were shown in Table 5. Three presenting features of Autism were significantly observed to be higher among boys with at high risk of ASD than among girls. Behavioral/Emotional expression problems were significantly two times higher among boys with at high risk of ASD than among girls with at high risk of ASD (COR = 1.96, CI: 1.32–2.91). Whereas, Reduced peer interaction and insistence of children to play with the same thing the same way were significantly more than one and a half times higher among boys with at high risk of ASD than among girls with at high risk of ASD (COR = 1.64, CI: 1.22–2.21 & COR = 1.61, CI: 1.17- 2.19). The odds of having poor to limited comprehension/Limited language/nonverbal, speech difficulties, learning, and academic performance difficulties were insignificantly higher among boys than girls.

**Table 5:** Odds of presenting features of Autism among surveyed children according to sex

**Presenting features of Autism**^a^	**All ASD** **(*n* = 1370)** **n(%)**	**Boys** **(*n* = 879)** **n(%)**	**Girls** ***n* = 491** **n(%)**	**COR (95% CI) between boys and girls**
Child does not usually express joy when seeing parents (Behavioral/Emotional expression problems) (*n* = 257)	150 (58.4)	115 (44.7)	35 (13.7)	1.96 (1.32–2.91)**
Child does not play with mates and do not socialize (Reduced peer interaction) (*n* = 450)	262 (58.2)	191 (42.4)	71 (15.8)	1.64 (1.22–2.21)**
Child insists to play with the same thing the same way (*n* = 431)	238 (55.2)	173 (40.1)	65 (15.1)	1.61 (1.17- 2.19)**
Child is beyond his/her mates in comprehension (Poor to limited comprehension/Limited language/nonverbal) (*n* = 886)	379 (42.8)	252 (28.4)	127 (14.4)	1.15 (0.89–1.48)
Child is beyond his/her mates in speech (Speech Difficulties) (*n* = 1627)	671 (42.0)	455 (28.0)	216 (14.0)	1.37 (0.31–1.71)
Child is beyond mates in school (if he/she is in school)^b^ (learning and academic performance difficulties) (*n* = 544)	149 (27.4)	101 (18.6)	48 (8.8)	1.9 (0.83–1.72)

^a^percent was calculated out of rows

^b^calculated out of school aged children (6–12 years)

^**^p-value highly sig at < 0.01

Twenty four significant variables associated with children who screened + ve for at high risk of ASD versus those who screened negative of the healthy population were entered into a multivariate logistic regression using the enter selection procedure (Table 6). The odds of being at high risk of autism increased significantly with being males by almost one and three-quarters times that being females (AOR = 1.70, 95%, CI:1.51–1.92).

**Table 6:** Multivariate Logistic regression model for predictor ASD

Parameters	Screen + ve ASD total Vs. Screen -ve ASD total		
	**Wald**	**OR**	**CI**
Age	.051	1.00	0.98- 1.02
Sex	75.95	1.70	1.51–1.92*
No of households	2.51	0.95	0.89- 1.01
Locality (rural)	4.902	0.84	0.73–0.98*
Social level (Middle Vs. Low)	5.708	1.76	1.52–2.03*
Social level (High to low)	58.10	1.20	1.03–1.40*
Residency (Lower to cities)	68.38	0.47	0.39–0.56*
Residency (Upper to cities)	1.87	0.98	0.96–1.08
Residency (Frontiers to cities)	46.94	0.46	0.35–0.56*
Maternal age at birth	.080	1.00	0.99–1.01
Maternal University and above to less	10.54	0.66	0.52–0.85*
Paternal University and above to less	13.37	0.64	0.51–0.82*
School attending status (not)^a^	4.43	1.22	0.92–1.28
Maternal work status (working)	1.31	0.90	0.75–1.08
Mothers have any health problems during pregnancy	34.74	1.74	1.45 – 2.10*
Difficult labor	6.96	1.24	1.06–1.45*
Disabled mothers	4.78	1.35	0.92–1.50
Less than 7 months (preterm delivery)	1.90	0.89	0.98–1.94
LBW (less than 2.5 kg at birth)	14.80	1.53	1.23–1.89*
Children suffer from jaundice after birth	7.26	1.20	1.05–1.37*
Children suffer from cyanosis after birth	14.38	1.87	1.35–2.59*
Children suffer from any convulsions	89.01	3.67	2.80- 4.80*
Children kept in an incubator for more than two days	16.62	1.47	1.22–1.76*
Child suffer from meningitis	.223	0.9	0.58–1.40
Constant	138.96	0.043	

^*^p-value significant at < 0.05

^**^p-value highly sig at < 0.01

^a^of children aged 6 -12 years

The neonatal problems related predictors for at high risk of ASD in order were: Children with a history of any convulsions increased the odds of autism by almost four times compared to those not having convulsions (AOR = 3.67; 95% CI: 2.8–4.8), children with a history of cyanosis after birth (AOR = 1.87; 95% CI: 1.35–2.59), history of LBW babies (AOR = 1.53; 95% CI: 1.23–1.89). Children kept in an incubator for more than two days (AOR = 1.47; 95% CI: 1.22–1.76) were associated with increased odds of being at high risk of autism by nearly one and a half times compared to healthy and full-term born children.

Maternal predictors were having either a history of health problems during pregnancy or a history of difficult labor. They increased the odds to be at high risk of autism by one and half times compared to healthy mothers (AOR = 1.74; 95%, CI: 1.45–2.10 & AOR = 1.74; 95%, CI:1.45–2.10). Maternal factors are not age-specific predictors.

The protective factors that decrease the odds to be at high risk of autism included: being resident in a rural locality with a decrease in the odds to be at high risk of autism by 15% compared to being resident in urban communities (AOR = 0.84, 95%, CI: 0.73 – 0.98), maternal and paternal education with university or above degree decrease the odds to be at high risk of autism by approximately 40% compared of being with less degree of education (AOR = 0.66, 95%, CI: 0.52 – 0.85 & AOR = 0.64, 95%, CI: 0.51– 0.82 respectively). Being resident in frontiers or in Lower Egypt decreases the odds of being at high risk of autism by almost 50% compared to being resident in cities (AOR = 1.5, 95%, CI: 1.09 – 2.06).

## Discussion

ASD is a complex developmental disorder that causes a burden to affected children and their families. Early detection and intervention lead to a better prognosis with decreased psychological and economic burdens on the families and society. According to Leo Kanner, the first prevalence estimate of ASD was reported in 1966 to be 4.5 per 10,000 people among children aged 8 to 10 in the United States [51]. Meanwhile, there is a consensus perception about the increase in ASD prevalence worldwide over the last decade. This perception may be attributed to the increased public awareness and better diagnostic services [4], earlier autism diagnosis, and/ or the discovery of therapeutic targets [88]. Accordingly, the current study focused on screening to detect the at high risk of autism with their risk factors. This step is important at the primary health care level to detect suspicious cases for early management. The current study was both a community and facility-based screening study for detecting children at high risk for ASD and determining the risk factors among 41,640 children aged 1–12 years.

There is a growing increase in ASD prevalence worldwide. However, the prevalence is variable in different countries; in the USA, 1 in 59 children had ASD [12] and in the UK, the prevalence of ASD was 1.1% [102]. Few researchers have looked at the national prevalence of ASD among Arab countries as estimated by a recent systematic scoping review [2] with the reported lesser prevalence of ASD than that reported among the developed countries. In Arab countries, it is reported that there is a wide range in prevalence of ASD from 0.014 to 4.7% [2]. The estimate was 2.9 per 1000 in the United Arab Emirates [102], 1.4 per 10,000 in Oman, 4.3 per 10,000 in Bahrain, and 1/167 in Saudi Arabia [5, 81]. The estimated variations in ASD prevalence between countries were mainly attributed to the methodological tools used for the autism diagnosis. It is well known that the confirmation of ASD diagnosis is usually done by specialized clinicians using both DSM5 (Diagnostic and Statistical Manual of Mental Disorders (DSM-5), [23] and CARS [84]. Accordingly, the terminology used for defining autism prevalence should be very precise according to the used tool for diagnosis. Another contributing factor to the detected variation was the targeted age group differences between studies as well as the level at which the study was conducted; whether at the community or facility level.

Few studies were done in Egypt to detect either the prevalence of the at high risk of Autism or ASD confirmed cases; mostly clinic-based, typically with small sample sizes, and were not representative of the whole country. Although ASD is frequently underdiagnosed or misdiagnosed in Egypt [2], existing studies were suggestive that ASD is a prevalent disorder in Egypt. The majority of prevalence studies in Egypt included samples from specialized units or hospitals and were related to the confined age group as a target group. The main objective of the current study was reporting the real situation of children at high risk of ASD as a national screening estimate through two screening phases. The first phase was a community-based one through using Vineland Adaptive Behavior Scales to detect children suspicious for autism. The second screening phase was a facility-based for reporting the prevalence of the high risk of ASD among the referred suspicious children aged 1–12 years using the M-CHAT for children aged 1–3 years, GARS-2 for children 3–12 years. In addition, Denver II Developmental screening test for children up to 6 years was used to confirm developmental delay detected in phase I screening and documenting possible developmental regression that is affected because of autism. The current study estimated the prevalence of the at high risk for autism to be 3.3% (95% CI: 3.1%–3.5%). This estimate was in accordance with two facility-based Egyptian studies. The first study investigated 500 children aged from 3 to 12 years in one Egyptian governorate according to GARS-2 and showed the prevalence of ASD to be 3.4% [11]. The other Egyptian screening study was done in kindergartens on 3722 preschool children and estimated that 2.8% of children were at high risk for ASD according to Modified Check List for Toddlers/Revised (M-CHAT-R) [102].

The current study reported that the highest prevalence was for the age group 5- < 6 years (6.4%, 95% CI: 5.7%–7.2%) followed by age group 6- < 7 years (5.7%, 95% CI: 5.0%–6.5%). The study revealed also that children aged 3 – < 6 years were the most likely to be diagnosed with ASD (4.4%). This was in accordance with another study which found that the most common age of presentation was between 2–5 years of age [103]. This finding supports the crucial recommendation for the early detection of ASD in preschool children [49, 54]. Unfortunately, the majority of children with ASD do not obtain a diagnosis until they reach school age. The fact that many physicians- especially in rural communities- have insufficient awareness of the diagnosis of ASD resulted in a delay in referral of the at-risk children to ASD specialists, another reason was the lack of awareness of mothers about the developmental milestones until their children reach school age [86]. This values the importance of screening at the community level to provide a real situation for ASD problems.

The present study's finding of having that ASD among boys was one and three-quarters times more than that of girls in the age range 1–12 years (OR 1.72, 95% CI: 1.53%–1.92%) was in parallel with many studies that revealed a higher frequency of ASD among males than females with a ratio of 2:1 [3], 3:1 [11, 51], or 4:1 [50, 67]. Results of different studies have been contradictory. When screening the entire population using gold standard assessments, current estimates suggest around 3 males receive an autism diagnosis for every female. However, in clinical samples who have already received an autism diagnosis, that ratio is higher at over four males to each female [50]. In individuals with intellectual disability, the ratio is closer to 2:1 [101].

One of the first signs of ASD is delayed speech. However, the presence of speech before the age of five is the most powerful predictor of better improvement of ASD symptoms [69]. The odds of having speech difficulties, learning, and academic performance difficulties were insignificantly higher among boys than girls in the current study (COR = 1.37, CI: 0.31–1.71). Our results reported three presenting features of autism to be significantly higher among the at-risk boys than among the at-risk girls for ASD. Behavioral and emotional expression problems were significantly associated with nearly two times higher odds of ASD risk among boys than among girls (COR = 1.96, CI: 1.32–2.91). Whereas, reduced peer interaction and insistence of children to play with the same thing the same way carried significantly more than one and a half times higher odds among boys than among girls with at risk of ASD (COR = 1.64, CI: 1.22–2.21 & COR = 1.61, CI: 1.17- 2.19). Our study comes in agreement with Yousef and his colleagues [102] who reported behavioral disturbance, significant developmental and speech delay among ASD patients, also with another Egyptian study that was done by El-Baz and his colleagues who proved that ASD is associated with developmental delay, speech defect, psychological disorders, and intellectual and learning disabilities [28, 40, 45, 102]. Alshaban and his coworkers [8] reported that 75.1% of children with ASD had language delays for words, 91.4% for phrase speaking, and 19.4% had developmental regression. They also reported that 19.4% of children had persistent deficiencies in expressive language and 14.0% in peer interactions.

There have been not enough evaluations on sex differences by ASD end phenotypes to date. However, some researchers reported that males with ASD are more likely than females to suffer from repetitive behaviors and have limited interests [17, 39, 78]. The odds of having poor to limited comprehension or limited language or nonverbal difficulties were insignificantly higher among boys than girls in the present study. This finding was also similar to other researchers who found no significant differences between males and females with ASD in communication, comprehension difficulties, or language skills [71, 89, 91].

ASD risk factors have yet to be properly defined [102]. The current study identified the determinants of children who were at high risk for ASD that are related to socio-demographic, epidemiological, maternal, and child health status. Cultural aspects of ASD remain understudied. Saudi Arabia and Lebanon provided the majority of the studies and declared no association between ASD and socioeconomic status [25, 32]. Investigating the association between parental socioeconomic status and risk of ASD is important to be clarified as it could provide insight into the etiology of ASDs, which is still largely not understood [75]. Some investigators have found a strong link between ASD and urbanity (i.e., a higher risk of autism in urban than rural districts) [48, 77, 98]. Our results revealed the same and reported that living in Lower Egypt or in Frontiers significantly decreased the odds at risk of ASD than living in urban cities by more than 60% (OR = 0.39, CI: 0.34–0.44 & OR = 0.36, CI: 0.29–0.45 respectively). Moreover, the at-risk of ASD was significantly higher among the middle class than in both the high and low classes (OR 1.53, 95% CI: 1.34–1.73 & OR 1.72, 95% CI: 1.51–1.97 respectively). Many studies implicated the role of the socioeconomic status of parents in the early diagnosis of ASD as a low socioeconomic class may delay taking medical advice and this could lead to the child not being probably diagnosed [53, 54]. However, in the current study the association between the middle class and the risk of ASD could be attributed to the lack of functional role of the parents especially mothers. The majority of mothers belonging to the middle class are working for a long time, seldom communicating with their children (play, talk or teach things) being overwhelmed by the home duties, besides their limited awareness about children's developmental milestones. Most of children belonging to the middle class have been constrained for screens for prolonged periods of time. A recent study showed that longer durations of screen time among 1-year-old boys was significantly associated with ASD at 3 years old [47]. On the other hand, women belonging to the low social class leave their children to play outside the home with their peers or mates which had a positive impact on the improvement of their cognitive and social skills. Whereas, children belonging to the high class often regularly attend a preschool education program that enhances children's communication skills.

Systematic review studies revealed that advanced paternal and maternal age has been associated with the risk for autism [52, 55, 74].

When looking at the odds of having ASD, mothers who were over 35 years old at the time of birth carried one-and-a-half- higher odds of risk for ASD compared to mothers who were under 18 years old (OR = 1.57, 95 percent CI: 1.13–2.18) in the current study.

The present study revealed that children with mothers or fathers who had higher education were less likely to be associated with a risk of ASD, especially those who had a college or greater education level with a fewer odds of a ranging from 25 to 39% than lower grades of education indicating that education serves as a protective factor. This could be supported by many Egyptian studies which concluded that awareness of the mothers regarding developmental milestones [27, 64, 79] and their children feeding practices [61] increased with the attainment of maternal education.

Unfortunately, this finding was in contrast with other Egyptian studies for which the majority of ASD children's moms were university graduates [29, 102], A Saudi Arabian study discovered also a high educational level of ASD children's moms [9]. Such variability in the mentioned studies could be attributed to the selection criteria with the exclusion of illiterate parents and those of a low education level from participating due to their negligence in filling the checklist questionnaire. These studies declared their selection as one of their studies’ limitations.

Until now no obvious causes for ASD could be fully understood, no single factor is responsible for the development of ASD but some multifactorial determinants seem to operate in the development of this disorder. On looking up predictors of the at high risk for ASD, prenatal and postnatal insults showed a significant impact. Children with a history of convulsions were associated with increased odds of being at high risk for autism by almost four times compared to those not having convulsions (AOR = 3.67; 95% CI: 2.8–4.8). It has been estimated that between 15 to 47% of people with ASD have epilepsy in early childhood [46].

Meanwhile, children with a history of cyanosis after birth or with a history of LBW or those who were kept in an incubator for more than two days or with a history of LBW babies were associated with increased odds to be at high risk for ASD by nearly one and a half times compared to healthy and full-term born children. The history of maternal health problems during pregnancy or difficult labor was associated with increased odds to be at high risk of ASD by one and half times compared to healthy mothers (AOR = 1.74; 95%, CI:1.45–2.10 & AOR = 1.74; 95%, CI:1.45–2.10). This finding supports other studies that affirmed the significant implication of perinatal insults for the development of ASD [11, 14]. Our study supports the recommendation that policymakers have to be concerned with the provision of services mainly during the perinatal period. In Egypt, community and health facilities based interventions had a great impact on reducing maternal and neonatal adverse outcomes [65, 58].

## Strengths

Our study has some important strengths. To the best of our knowledge, this study is considered to be the first study to investigate the prevalence and predictors for children who are at high risk of ASD at a national level with a very large sample size of Egyptian children aged 1–12 years and as a representative to Egypt. Accordingly, we could make it easy to generalize our findings. The use of two phases for screening using reliable screening tools is a strength. Our study also was the first that determined sex-specific estimates and predictors of ASD.

## Limitations

The lack of studying the environmental variability in the demographic status of the participating children is a weakness of this study. Environmental factors such as industrially emitted heavy metals, chemicals, bacterial, and viral infections may all play a role in ASD. The documented impact of environmental education and awareness-raising at the community level in Egypt in having a profound effect on knowledge attainment was proved [62, 63]. Out of limitation was studying the influence of childhood malnutrition which may be risk factors for ASD, especially the impact of school feeding programs on improving children’s growth and development was documented also in Egypt [60, 80]. The last-mentioned two factors are limited to be investigated during screening studies because of the long time required for filling the related questionnaires.

## Conclusion and recommendation

In this paper, we focused on the whole community to determine the prevalence of children who are at high risk of ASD among 41,640 children aged 1 to 12 years in Egypt through community-based national screening. This study was conducted among 8 Governorates representing all geographic regions of Egypt. The survey highlighted the most important contribution to the need for community-driven data to detect the risk and the protective determinants of ASD in general and among sex which when neglected can lead to a severe degree of ASD that could impact not only the life of the children but the whole family. Most of the detected determinants were preventable factors indicating that the educational and rehabilitation fields are far beyond the medical ones. Thus identifying the prevalence and the risk factors for the high risk of ASD is a step toward implementing surveillance for the at-risk of autism which is drastically needed for creating awareness among health care workers and caregivers. Neglecting to study the extent of the problem of the spread of autism makes more burdens on the families and the governments and helps in increasing the rate of disabilities.

The study findings necessitate prompt actions of local health and educational systems to be appropriately equipped to support affected children and their families optimally. Local health units should be provided with multidisciplinary teams including developmental and behavioral pediatrician, psychiatrist, psychologist, speech therapist, physiotherapist and social worker for early detection, proper diagnosis and management of these children. In addition, efforts must be taken to reduce the uncertainty around the proper estimates of this lifelong disorder. These efforts may include; screening of all children for ASD at ages 9, 18 and 24 months, along with regular developmental surveillance during vaccination visits, screening of preschool and school age children for ASD on admission to kindergarten and primary schools and consistent courses for primary physicians, school teachers, and kindergarten supervisors about early warning signs of ASD.

## Supplementary Information

**Additional file 1: S Table-1.** (List of the targeted Households (HH) according to the governorates, locality and sociodemographic status for screening of at high risk autism among children aged 1-12 years).

**Additional file 2: S Table-2.** Raw data (XLSX) that are anonymous.

## Data Availability

The datasets used and analyzed during the current study are anonymous and are fully available without restriction as S Table-2 Raw data (XLSX).

## Abbreviations

AOR: Adjusted Odds Ratio
ASD: Autism Spectrum Disorder
CAPMS: Central Agency for Public Mobilization and Statistics
CARS: The Childhood Autism Rating Scale
CDC: Centers for Disease Control and Prevention
CI: Confidence Interval
COR: Crude Odds Ratio
DDs: Developmental delays
DDST: Denver II Developmental Screening Test
DSM-5: Diagnostic and Statistical Manual of Mental Disorders (5th edition)
ERF: Economic Research Forum
GARS-2: Gilliam Autism Rating Scale
LBW: Low Birth Weight
M-CHAT-R/F: Modified Checklist for Autism in Toddlers-Revised
MOHP: Ministry of Health and Population
NRC: National Research Centre of Egypt
SD: Standard Deviation
SPSS: Statistical Package for the Social Sciences
UK: United Kingdom
USA: United States of America
VABSA: Arabic version of Vineland Adaptive Behavior Scales
WHO: World Health Organization
